# π‐Complexes of Diborynes with Main Group Atoms

**DOI:** 10.1002/asia.202000185

**Published:** 2020-04-16

**Authors:** William C. Ewing, Theresa Dellermann, Y. T. Angel Wong, James D. Mattock, Alfredo Vargas, David L. Bryce, Rian D. Dewhurst, Holger Braunschweig

**Affiliations:** ^1^ Institute for Inorganic Chemistry Julius-Maximilians-Universität Würzburg Am Hubland 97074 Würzburg Germany; ^2^ Institute for Sustainable Chemistry & Catalysis with Boron Julius-Maximilians-Universität Würzburg Am Hubland 97074 Würzburg Germany; ^3^ Department of Chemistry and Biomolecular Sciences University of Ottawa Ottawa Ontario K1N 6N5 Canada; ^4^ Department of Chemistry School of Life Sciences University of Sussex Brighton BN1 9QJ Sussex UK

**Keywords:** boron, main group elements, solid-state NMR, π interactions, multiple bonds

## Abstract

We present herein an in‐depth study of complexes in which a molecule containing a boron‐boron triple bond is bound to tellurate cations. The analysis allows the description of these salts as true π complexes between the B−B triple bond and the tellurium center. These complexes thus extend the well‐known Dewar‐Chatt‐Duncanson model of bonding to compounds made up solely of p block elements. Structural, spectroscopic and computational evidence is offered to argue that a set of recently reported heterocycles consisting of phenyltellurium cations complexed to diborynes bear all the hallmarks of π‐complexes in the π‐complex/metallacycle continuum envisioned by Joseph Chatt. Described as such, these compounds are unique in representing the extreme of a metal‐free continuum with conventional unsaturated three‐membered rings (cyclopropenes, azirenes, borirenes) occupying the opposite end.

## Graphical abstract

**Side‐on complexes** between alkynes and transition metals are commonly described as π‐complexes, metallacycles, or somewhere in between. Described here, a set of recently reported three‐membered heterocycles comprised of a diboryne complexed to phenyltellurium cations represent a historically elusive example of a π‐complex consisting solely of p‐block elements.

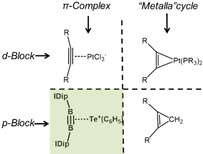

The side‐on complexation of alkenes and alkynes to transition metals is generally described in the language of the Dewar‐Chatt‐Duncanson (DCD) bonding model^1^, ^2^ as an interplay between σ‐donation from the π‐system of the multiple bond and π‐symmetry backdonation from filled d‐orbitals on the metal to vacant π* orbitals on the multiple bond. In 1957, Joseph Chatt discussed the use of this model as it pertains to the complexation of acetylenes to platinum.^3^ Vibrational spectroscopy performed on the studied complexes, triple‐bond adducts of [Pt(PPh_3_)_2_], hinted at sizeable reductions in the bond orders of the bound acetylenes. After noting this, Chatt discussed two possible bonding schemes, a donation/backdonation model where the contribution of the backdonation (d_Pt_→π*_C≡C_) outweighed the σ‐donation of the alkyne (π_C≡C_→d_Pt_) and a metallacyclopropene model featuring two Pt−C two‐center‐two‐electron bonds with a formal double bond between the carbon atoms. Though he favored the latter, he deftly hedged his bet by commenting that the orbitals existed for “one structure to take on part of the character of the other”. In doing so, he allowed for the idea that the bonding in Pt‐alkyne complexes, and hence all such metal‐alkyne complexes, may fall on a continuum ranging between pure π‐complexes, where the bonding is primarily π_(C≡C)_→d_(M)_ in nature with negligible backdonation, and metallacycles where backdonation dominates and the orbitals at the alkynyl carbon may be appropriately viewed as rehybridized toward the formation of two new C−M bonds.

Figure 1 depicts a few examples of organometallic compounds favoring either the π‐complex description or the metallacyclic formulation. The π‐complexes described here are generally the result of the bonding of alkynes to high‐valent (e. g., Pt^2+^, Au^3+^)^4^, ^5^ metals or compact, hard metals (Ag^+^)^6^ unlikely to redistribute d‐electrons. The metallacycles^3^, ^7^, ^8^, ^9^ are alternatively composed of electron rich, low‐valent metals, which readily participate in d_(M)_→π*_(C≡C)_ backbonding.

**Figure 1:**
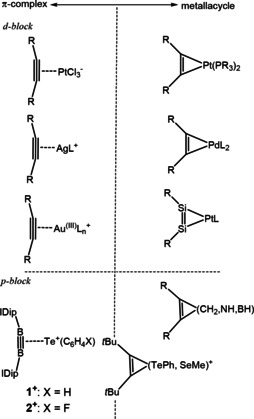
Examples of three‐membered cyclics exhibiting either π‐complex or metallacyclic characteristics. IDip=1,3‐di(2,6‐di*iso*propylphenyl)imidazol‐2‐ylidene. R=alkyl, aryl, H. See text for related references.

Despite the current ubiquity of the continuum model for describing organometallic cyclics, it is not frequently employed to describe metal‐free systems. The smallest unsaturated three‐membered rings (cyclopropenes, azirenes, borirenes) are instead most commonly described by valence bond theory as the product of overlapping hybrid orbitals, bent so as to accommodate the imposed geometry of the small ring.^10^ Though useful in practice and pedagogy, this model is not without its drawbacks, and as such, alternative views on the bonding environment have been offered ranging from σ‐bond delocalization^11^ to π‐bridged σ‐bonding.^12^ Though lacking accessible d‐orbitals with which to participate in d_(M)_→π*_(C≡C)_ backbonding, main group elements in such unsaturated three membered rings have a p‐orbital lying in the plane of the adjacent multiple bond with the correct symmetry for p→π* backbonding.^13^ If viewed as part of the DCD‐continuum, small rings such as cyclopropene would constitute the extreme re‐hybridized end of the spectrum; however, such a supposition requires examples of the other extreme, a metal‐free π‐complex where the bound alkyne maintains the majority of its triple‐bond character.

A number of recent examples have emerged of three‐membered heterocycles formed via the reaction of elemental chalcogens with disilenes (Si=Si double bonds) with structural features eliciting their description as π‐complexes,^14^ but such complexes formed with triple bonds are still unknown. Upon complexation to neutral metals (Pt and Pd), disilynes (RSi≡SiR) perhaps unsurprisingly form compounds identified as metallacycles by the substantial structural differences between bound and free disilyne.^9^ The closest examples of metal‐free alkynyl π‐complexes come from the complexation of bulky acetylenes to organo‐tellurirenium and ‐selenirenium fragments,^15^ but even in these cases, as will be discussed below, the geometrical features of the compounds put them somewhere between the π‐complex and rehybridized extremes of the DCD continuum.

We recently reported the syntheses of two new three‐membered heterocycles formed in the reactions of diborynes with diarylditellurides (Figure 1, bottom left).^16^ These compounds, in a departure from conventional metal‐free cyclics, clearly displayed the signatures of bonding dominated by a π_(B≡B)_→Te donation. The first hints were provided by X‐ray crystallography (Figure 2a). Structurally, a π‐complex in the DCD model would show limited differences between the free and bound triple bond, while a metallacyclic structure would evidence significant elongation of the triple bond (Δ*d*=*d′*−*d*, Figure 2b) and substantial *cis*‐bending away from the linear structure (α, Figure 2b). When the distances between the boron atoms in **1** and **2** were measured (**1**, 1.490(6) Å; **2**, 1.494(10) Å) and compared to the same distance in the uncomplexed diboryne (1.449(3) Å),^17^ difference (Δ*d*) values of 0.041 Å (**1**) and, 0.045 Å (**2**) were obtained. Figure 1c compares these values (using **1** only, due to the similarity of **1** and **2**) to other relevant complexes. As Pt^2+^ and Pt^0^ compounds have frequently been used as examples demonstrating the differences between π‐complex (Pt^2+^) or metallacyclic (Pt^0^) bonding, examples of both are also included in Figure 1c. While Pt^2+^ complexes of di‐*tert*‐butylacetylene such as [PtCl_2_(toluidine)(C_2_ *t*Bu_2_)] and [PtCl_3_(C_2_ *t*Bu_2_)]^−^ showed limited elongation of the C≡C bond (3.2%) in comparison to the much more drastic elongation of the C≡C bond in the zerovalent platinum complex [Pt(C_2_Ph_2_)(PPh_3_)_2_] (10.5%), the elongation found in **1** (2.8%) was even less. The bend‐back angles (α) tell the same story with the Pt^2+^ compounds showing comparatively mild bending (15°–20°) compared to the Pt^0^ species (40°–41°). The angles measured for **1** (15° and 16°) again fully support assignment as a π‐complex.

**Figure 2:**
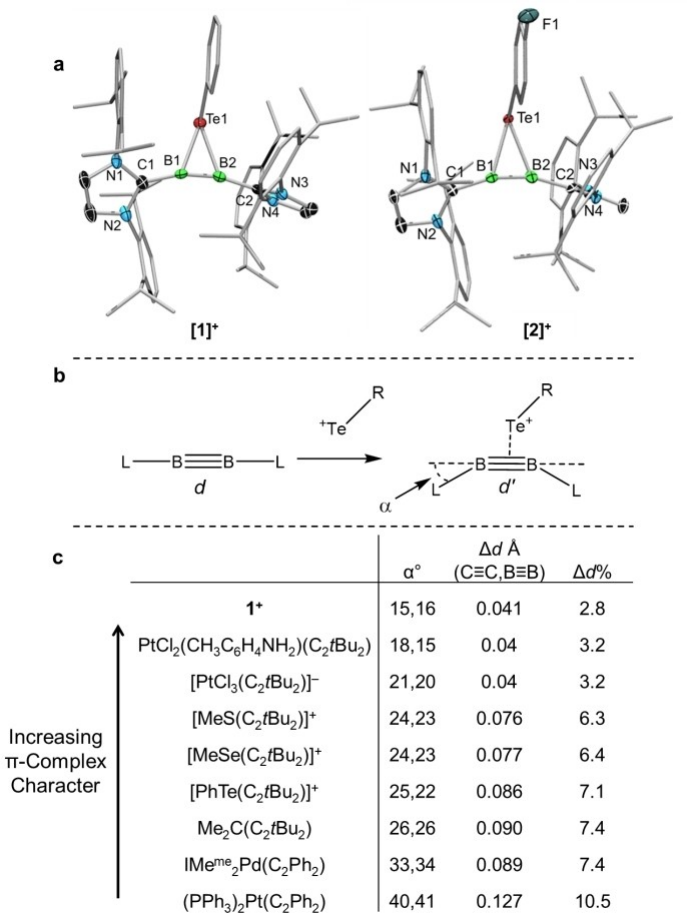
a) Solid‐state structure of cationic portions of **1** and **2**. Thermal ellipsoids represent 50% probability, and have been omitted from the ligand periphery. For clarity, all hydrogen atoms are likewise omitted. Selected bond lengths (Å) and angles (°): (**1**) B1–B2 1.490(6), C1−B1−B2 165.0(4), C2−B2−B1 164.1(3); (**2**) B1−B2 1.494(10), C1−B1−B2 164.7(7), C2−B2−B1 163.6(7). b) Structural metrics relevant to complexation in the DCD model. c) Table of structural measures of compounds on the π‐complex/metallacycle continuum. IMe^Me^=1,3,4,5‐tetramethylimidazol‐2‐ylidene. For references, see text.

These measures in other metal‐free systems formed of chalcogens and C_2_ *t*Bu_2_ 18 such as thiirenium (α: 24°, 23°; Δd%: 6.3),^19^ selenirenium (α: 24°, 23°; Δd%: 6.4)^15^ and tellurirenium (α: 22°, 25°; Δd%: 7.1)^15^ ions show them to lie somewhere in the middle of the continuum and indeed further to an end established by cyclopropane (e. g., 1,2‐di‐*tert‐*butyl‐3,3‐dimethylcyclopropene,^20^ Figure 1c). The similarities between the all‐carbon ring and the chalcogen‐containing heterocycles, when compared to **1^+^**, underscore the importance and uniqueness of the B≡B bond in the stabilization of such a π‐complex in the absence of a metal.

Vibrational spectroscopy is a second common way to evaluate π‐bound complexes within the DCD model. Large decreases in the vibrational frequencies (Δν_C≡C_) of triple bonds upon complexation are indicative of bond weakening through backdonation to a π* orbital and a decrease in bond order in the resulting metallacycle. Alternatively, small decreases are a hallmark of π‐complexes, as they signify only minor perturbation to the triple bond. The Δν_C≡C_ values for alkynes coordinated to platinum are diagnostic, as the C_2_ *t*Bu_2_ bound to Pt^2+^ ([PtCl_3_(C_2_ *t*Bu_2_)]^−^, Table 1) shows a mild decrease of only ∼200 cm^−1^ upon complexation, whereas the decrease observed in the complexation of C_2_Ph_2_ in the formation of [Pt(C_2_Ph_2_)(PPh_3_)_2_] is more than double (∼450 cm^−1^). The Raman spectra of **1** and **2** show even less significant changes in their central triple bonds (Δν_B≡B_=∼120 cm^−1^) than observed in the Pt^2+^ complexes.^16^, ^20^ Once again, these small differences stand in contrast to the complexation of a heavy chalcogen fragment (MeSe^+^) to a bulky alkyne, as the complexation of diadamantylacetylene results in a ν_C≡C_ value of ∼380 cm^−1^ in the final selenirenium ion.^15^

**Table 1:** The vibrational and NMR characteristics of compounds **1** and **2** in comparison to other relevant species. Ad=adamantyl; duryl=2,3,5,6‐tetramethylphenyl; IMe=1,3‐dimethylimidazol‐2‐ylidene; Th(TMS)=5‐(Me_3_Si)C_4_H_2_S.

Vibrational Spectroscopy		NMR Spectroscopy		
Compound	Δν [cm^−1^]	Compound	*J*(^11^B,^11^B) [Hz]	%*s*‐orbital character
**1**	120^[a]^	**1**	171±1	49.3
**2**	120^[a]^	**2**	173±1	49.1
[PtCl_3_(C_2_ *t*Bu_2_)]^−^	200^[b]^	B_2_IDip_2_	187±5^[e]^	52.5
[MeSe(C_2_(Ad)_2_)]^+^	380^[c]^	B_2_(IMe)_2_(Duryl)_2_	85±10^[e]^	32.5
(PPh_3_)_2_Pt(C_2_Ph_2_)	450^[d]^	B_2_(IMe)_2_(Th(TMS))_2_	75±3^[e]^	31.5

[a] ref. 21. [b] ref. 22. [c] ref. 15. [d] ref. 23, 24. [e] ref. 26.

Recent work by Bryce and coworkers has established the utility of solid‐state NMR spectroscopy in gleaning information about molecules containing boron‐boron bonds,^25^ and in particular as a measure of bond order in diborenes, diborynes and related compounds.^26^ We previously reported on the observed *J*(^11^B,^11^B) coupling constant of the diboryne B_2_IDip_2_ obtained through ^11^B double‐quantum‐filtered (DQF) *J*‐resolved NMR experiments (187±5 Hz).^26^ When combined with natural localized molecular orbital (NLMO) modeling indicating that the σ‐bond linking the two boron atoms had 52.5% s‐orbital character, the sp‐hybridization of the two boron atoms was evident, consistent with its assignment as a triple bond. The same measurements carried out on a pair of diborenes yielded substantially smaller *J*(^11^B,^11^B) values, ranging from 75–85 Hz, while NLMO modeling indicated 31–33% s‐character (*sp* ^2^ hybridization). When this technique was applied to compounds **1** and **2** (Figure S1), values of 171±1 and 173±1 Hz were obtained, both strikingly similar to that of the free diboryne (Table 1). Subsequent NLMO modeling (Figure S2) indicated negligible rehybridization of the central boron atoms (∼49% *s*‐character), highly suggestive of an intact triple bond (Table 1). A more detailed summation of NMR experiments and NLMO modeling on several compounds for comparison is given in Table S1.

Each of these pieces of data indicating only mild perturbation to the B≡B triple bond upon coordination to the Te^+^ fragment support the assignment of **1** and **2** as metal‐free π‐complexes; however, the same evidence may be alternatively interpreted as evidence for binding through non‐covalent, electrostatic forces much like those reported by our laboratory in the coordination of alkali metals (Na^+^, Li^+^) to diborynes.^27^ Structural data points to dissimilarities in the bonding of the two types of compounds, as the B−Te bonds in **1** (2.260(4) Å, 2.247(4) Å) were found to be substantially shorter than those in either the diboryne‐Li^+^ (2.514(5) Å, 2.522(5) Å) or diboryne‐Na^+^ (2.764(2) Å, 2.768(2) Å) complex.^27^ Theoretical elucidation was sought through the use of Kohn‐Sham Density Functional Theory (DFT) calculations at the OLYP/ZORA/TZP level. Energy decomposition analysis (EDA, Table S2), carried out to determine the relative magnitudes of the electrostatic and covalent contributions to bonding, definitively showed the covalent portion (E_orb_=−253.03 kcal/mol) to be dominant over the electrostatic portion (E_el_=−189.19 kcal/mol). The difference in these two interactions may in part be explained by the electronegativity differences of the atoms in question. Boron and tellurium, with Pauling electronegativity values of 2.04 and 2.1, respectively, are more apt to form covalent interactions than is boron with the alkali metals (χ_Na_=0.93, χ_Li_=0.98).

Despite this difference, analysis of the charge flows in the molecule by means of the extended‐transition‐state natural orbitals for chemical valence (ETS‐NOCV) scheme yielded a similar story to the electrostatic bonding of alkali metals. When encountering an alkali metal ion, the diboryne is capable of concentrating electron density along the B≡B bond through the donation of π‐electron density from the stabilizing carbenes.^26^ Calculations on the cationic portion of **1** indicated the same to be true when covalently donating electrons to the Te fragment (π_B≡B_→p_Te_). Figures 3a and 3b depict the two most important electron‐flow channels based on the orbital interaction terms resulting from the interaction of the [PhTe]^+^ fragment with the diboryne. Areas in red indicate depletion of electron density, while blue shows areas of increased density upon combination of the fragments. For comparison, the same treatment was applied to the interactions between the more metallacyclic C_2_ *t*Bu_2_‐tellurirenium complex described above, with the two most important contributing interactions depicted in Figures 3c and 3d. In **1**, the more important of the two interactions is clearly the donation of electrons from the B≡B bond toward the [PhTe]^+^ fragment (π_B≡B_→*p*_Te_, −175.75 kcal/mol). The second of the two (Figure 3b) shows the movement of charge consistent with *p*_Te_→π*_B≡B_ backbonding, but it is energetically far less significant (−32.53 kcal/mol). Importantly, the calculation depicts electronic depopulation across the entire conjugated system stretching from the carbenes to the triple bond, indicative of the donation of electrons from the stabilizing carbenes in order to roughly maintain the electron density at the B≡B unit even without substantial backdonation. This is evidenced by the fact that the boron atoms retain their negative Hirshfeld charges, calculated to be −0.186 in the free diboryne and on average −0.118 in **1**, which are uncommon for electropositive elements bound to elements with greater electronegativities. The substantial lengthening of the C_carbene_−B bonds of **1** (1.552(5) Å, 1.551(5) Å) in comparison to the same bonds in B_2_IDip_2_ (1.487(3) Å, 1.495(3) Å) are a further indication of the depopulation of the conjugated system to enable π_B≡B_→Te_p_ donation.

**Figure 3:**
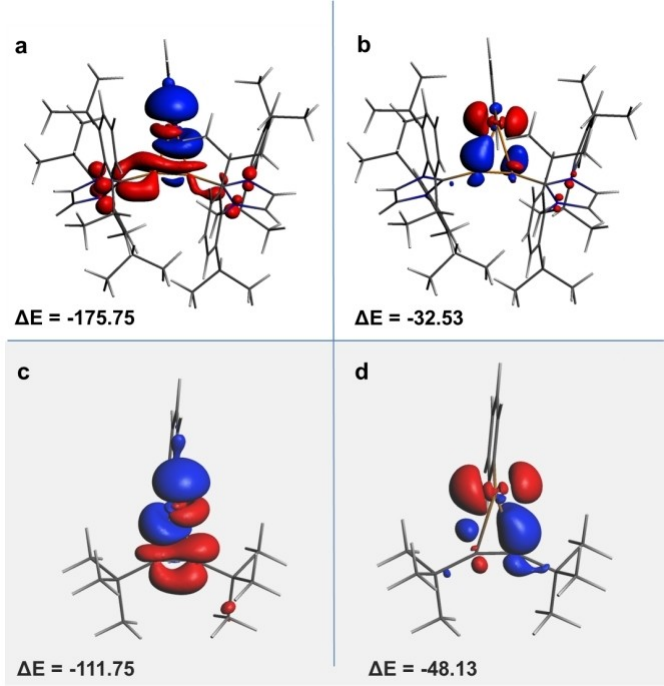
Contours of relevant deformation density of **1^+^** (a and b) and C_2_ *t*Bu_2_‐tellurirenium (c and d). Charge flow is from red to blue upon combination of the fragments. Fragments were defined as the free diboryne (B_2_IDip_2_) and phenyltellurium cation (PhTe^+^).

The same two types of charge flows were calculated for the interaction of [PhTe]^+^ with C_2_ *t*Bu_2_; however, in this complex the sigma donation is a weaker interaction overall, −111.75 kcal/mol versus −175.75 kcal/mol in **1**, and the backdonation is far more important. In **1**, backdonation only accounts for 15% of the total energy of the combined interactions, while instead constituting 30% of the interaction in the acetylene complex. The relative energetic unimportance of the Te_p_→π*_B≡B_ backdonation may be compared to that calculated for a hypothetical Ag^+^‐C_2_H_2_ complex (∼20%),^28^ to further underscore the degree to which compound **1** exemplifies Chatt's vision of the π‐complex.^29^

It was initially supposed that the ability of the B_2_‐unit to act strictly as a π‐ligand where the organic alkyne did not was enabled by both a relatively high‐energy HOMO (π_B≡B_) of the diboryne, making it a strong donor, and an energetic mismatch between the diboryne LUMO (π*_B≡B_) and the donating *p* orbital on Te.^23^ However, as Figure S3 shows, though the calculated HOMO and HOMO‐1 of the diboryne fragment are higher in energy than those of C_2_ *t*Bu_2_, their LUMO levels are roughly equivalent, meaning backdonation should be energetically possible in **1**. The fact that this does not happen must be attributed to the role of the electronic stabilization of the flanking N‐heterocyclic carbenes (NHCs) depicted in Figure 3a. In much of our work on (NHC)_2_B_2_ compounds^30^ we have shown how the characteristics and reactivities of the central B_2_ units were the result of the relative π‐acidities of the stabilizing NHCs, serving to attenuate the electron density at boron. In this case, the ability of the diboryne to covalently bond to the phenyltellurium cation as a π‐ligand is instead derived from the π‐basicity of the NHC. The conjugated π‐system acts as a conduit for electron density, and in doing so not only allows for the formation of a strong σ‐bond, but also inhibits deformation of the linear diboryne, which would accompany backbonding. In a more conventional vein, it is perhaps appropriate to view the B≡B bond as an exceptionally electron‐rich side‐on ligand, owing to its access to the carbene electrons, and hence unlikely to accept backdonation from Te.

In conclusion, we have provided evidence that compounds **1** and **2** represent metal‐free π‐complexes, aiding in the extension of the DCD model to three‐membered unsaturated heterocycles in the p‐block. It is conceptually appealing to be able to apply models of bonding as widely as possible across the periodic table, rather than relying on compartmentalized explanations of the behaviors of groupings of elements. It is our hope that by describing compounds **1** and **2** in language generally reserved for the d‐block elements to their left. We are taking positive steps in this direction.

## Conflict of interest

The authors declare no conflict of interest.

## Supporting information

As a service to our authors and readers, this journal provides supporting information supplied by the authors. Such materials are peer reviewed and may be re‐organized for online delivery, but are not copy‐edited or typeset. Technical support issues arising from supporting information (other than missing files) should be addressed to the authors.

Supplementary
