# Supplementary material for: π‐Complexes of Diborynes with Main Group Atoms
Source: Chem Asian J. 2020 Apr 16;15(10):1553–7. doi: 10.1002/asia.202000185 (PMC7317709; doi:10.1002/asia.202000185)
Supplement: Supplementary file 1 — Supplementary [file ASIA-15-1553-s001.pdf]

CHEMISTRY
AN ASIAN JOURNAL

Supporting Information

π-Complexes of Diborynes with Main Group Atoms

William C. Ewing, Theresa Dellermann, Y. T. Angel Wong, James D. Mattock, Alfredo Vargas, David L. Bryce, Rian D. Dewhurst, and Holger Braunschweig*© 2020 The Authors. Published by Wiley-VCH Verlag GmbH & Co. KGaA. This is an open access article under the terms of the Creative Commons Attribution License, which permits use, distribution and reproduction in any medium, provided the original work is properly cited.This manuscript is part of a special collection for the 20th Anniversary of the Tateshina Conference.

Solid-state NMR Experiments

All samples were packed into 4 mm o.d. ZrO_2 rotors under an argon atmosphere. The NMR experiments were performed using a Bruker AVANCE III 400 spectrometer with a Bruker triple resonance MAS probe. The ^{11}B DQF *J*-resolved spectra were recorded at 10 kHz MAS frequency with 20.2 μs central-transition selective 90° pulses and a 2 s recycle delay. A delay of 1.25 to 1.5 ms was used for the double quantum filter. High power ^{1}H decoupling was also implemented, and double frequency sweeps were employed for central-transition signal enhancement. In order to acquire the 2D spectra, 64 t_1 increments, with 256 scans per increment, were obtained with an incremented delay of 389.45 μs.

DFT Calculations for Bond Order Determination

For the $B_2(SIDip)_2$ sample, the published crystal structure was employed as the input structure without further optimization (CCDC number 1410969). For the $B_2(IDip)_2Te_2(Fp)_2$ and $B_2(IDip)_2Te_2(Ph)_2$ samples, the solvent molecules were removed and the isopropyl groups were replaced by protons in order to reduce computational time. The positions of the protons were then optimized using the Gaussian 09, rev. D.01.[1] and the PBE functional with the 3-21G basis set. DFT calculations of *J* couplings were then performed using the Amsterdam Density Functional program (ADF, ver. 2012 and ver. 2016).[2] The calculations were executed using the GGA functional with the TZP basis sets. ZORA was also implemented for the $B_2(IDip)_2Te_2(Fp)_2$ and $B_2(IDip)_2Te_2(Ph)_2$ samples. The NBO program (ver. 5.0 and ver. 6.0),[3-4] which is incorporated in ADF, was also employed in order to perform the NBO/NLMO analysis.

Table S1. The experimental and theoretical $J(^{11}B, ^{11}B)$ coupling constants acquired from the DQF *J*-resolved experiments and DFT calculations, respectively, and the s-character of the σ_{BB} bond obtained from the NLMO analysis for a set of compounds for comparison. Calculated values for **1** and **2** refer to calculations on the cations of the respective salts.

Sample	$J(^{11}B,^{11}B)_{exp.}$ [Hz]	$J(^{11}B,^{11}B)_{PBE/TZP}$ [Hz]	σ_{BB} s-character [%]
$[B_2(IDip)_2Te(Fp)^+][Te(Fp)^-]$ (**2**)	173 +/- 1	171.5	49.1
$[B_2(IDip)_2Te(Ph)^+][Te(Ph)^-]$ (**1**)	171 +/- 1	171.8	49.3
$B_2(IDip)_2$	187 +/- 5[a]	196.4[a]	52.5[a]
$B_2(CAAC)_2$	164 +/- 5[a]	167.8[a]	50.7[a]
$B_2(SIDip)_2$	189 +/- 1	193.2	56.4
$B_2(IMe)_2(Dur)_2$	85 +/- 5[a]	73.9[a]	32.5[a]
$B_2(IMe)(Th(SiMe)_3)_2$	75 +/- 5[a]	65.7[a]	31.5[a]

[a]Values obtained from reference 5.

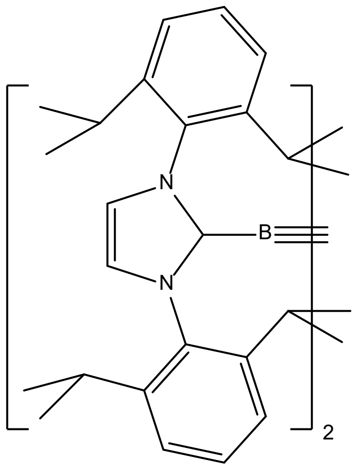

B_2IDip_2

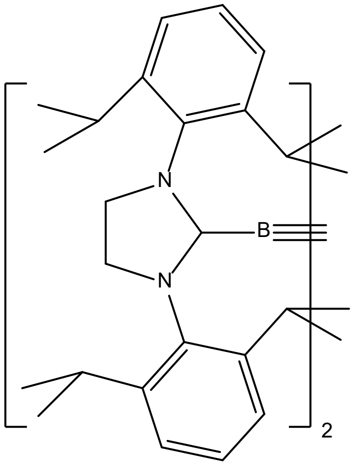

B_2SIDip_2

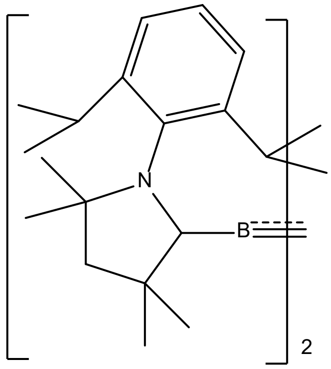

B_2CAAC_2

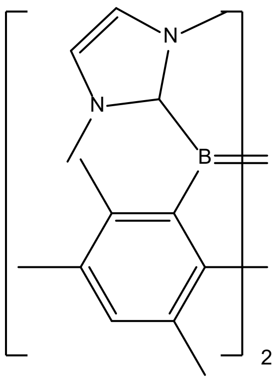

$B_2IMe_2Dur_2$

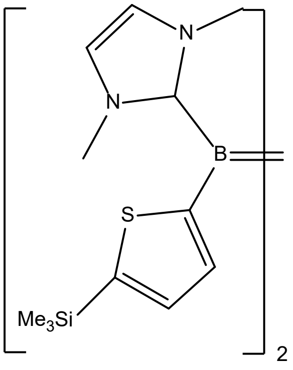

$B_2IMe_2(Th[SiMe_3])_2$

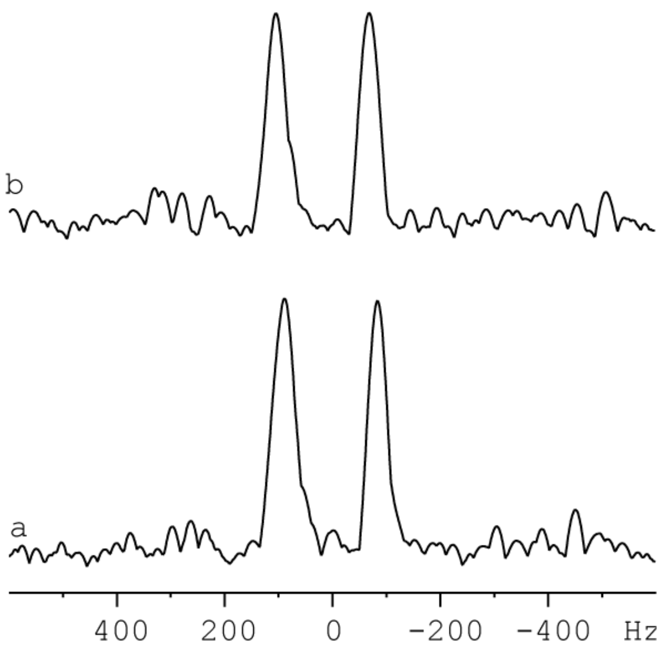

Figure S1. The indirect dimension of the ^{11}B DQF *J*-resolved spectra of (a) **2** and (b) **1**.

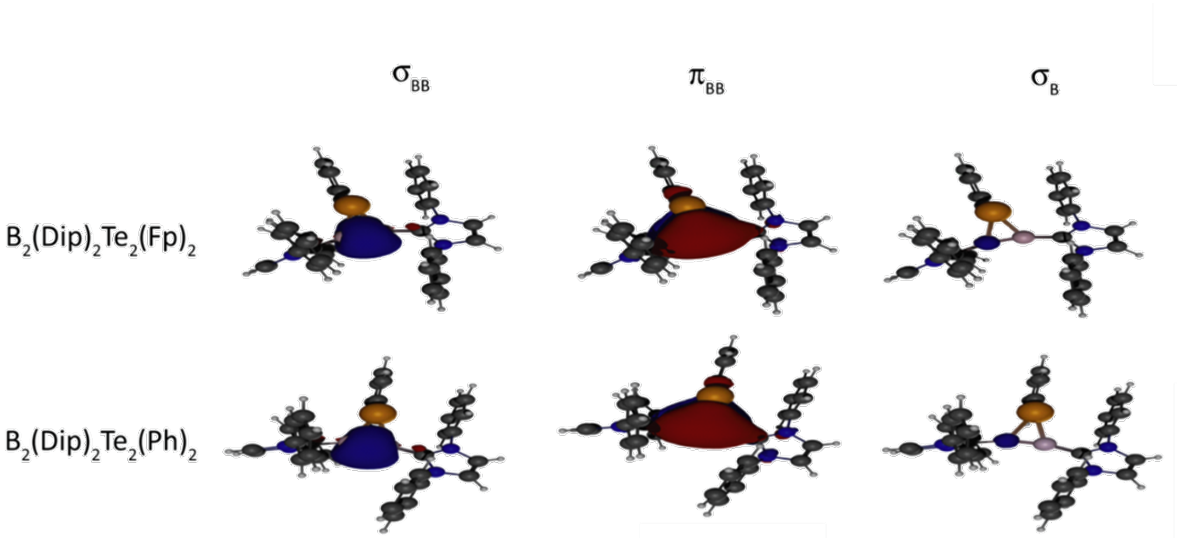

Figure S2. The NLMOs (σ_{BB}, π_{BB} and σ_B) of **2**$^+$ (top) and **1**$^+$ (bottom). The isopropyl groups were replaced by protons in order to reduce computational time.

Details for EDA and ETS-NOCV Calculations

Further calculations on **1⁺** and C_2tBu_2-tellurirenium were carried out using the Amsterdam Density Functional (ADF)[2] program at the OLYP/ZORA/TZP[6–10] level of theory. To obtain the singlet state, spin-restricted calculations were performed constraining the projection of the total electronic spin along a reference axis to 0. Frequency calculations were conducted to determine if each stationary point corresponds to a minimum.[11–13] Reported bond orders are of the Mayer bond order type[14,15] and atomic charges were determined according to the Hirshfeld charge analysis.[16,17] The nature of bonding is described using the energy decomposition analysis (EDA)[2,18] (also known as “fragment approach”) according to the methods of Morokuma[19] and Ziegler and Rauk.[20] Using the EDA scheme, the energy E_{int} associated to the interaction between a fragment (e.g. diboryne, alkyne) and another (e.g. phenyltellurate) can be divided into three components: $E_{int} = E_{elstat} + E_{Pauli} + E_{orb}$; the first term, E_{elstat}, corresponds to the classical electrostatic interaction between the unperturbed charge distributions of the fragments (the overall density being the superposition of the fragment densities). The second term, E_{Pauli}, expresses the energy change that arises upon going from the simple superposition of the fragment densities to the wavefunction that obeys the Pauli principle through antisymmetrization and normalization of the product of the fragment wavefunctions. In the last term, E_{orb}, the energy that originates from the contributions from stabilizing orbital interactions (electron pair bonding, charge transfer, polarization) is given. To further quantify the contributions of interactions between the fragments, we employed techniques based on the ETS-NOCV formalism. To describe the charge transfer, the natural orbitals for chemical valence (NOCV) description was used.[21,22] This is based on the NOCV wavefunction as an eigenvector of the deformation density matrix in the basis of fragment orbitals, the deformation density ($\Delta\rho$) is then decomposed in the NOCV representation, as a sum of pairs of complimentary eigenfunctions. A useful qualitative metric is the sign of $\Delta\rho$, negative for an outflow of charge and positive for an inflow of charge in going from the constituent fragments to the whole system. The Graphical User Interface (ADF-GUI) - a part of the ADF package, was used for visualization purposes.

Table S2. Results of an EDA assessment of compound **1⁺** and its alkynyl analog. For methods see Computational Details section above.

	**1^+**	C≡C/TeAr
E_{el}	–189.19	–135.97
E_{orb}	–259.03	–188.82
E_{Pauli}	272.76	222.67
E_{int}	–175.47	–102.13

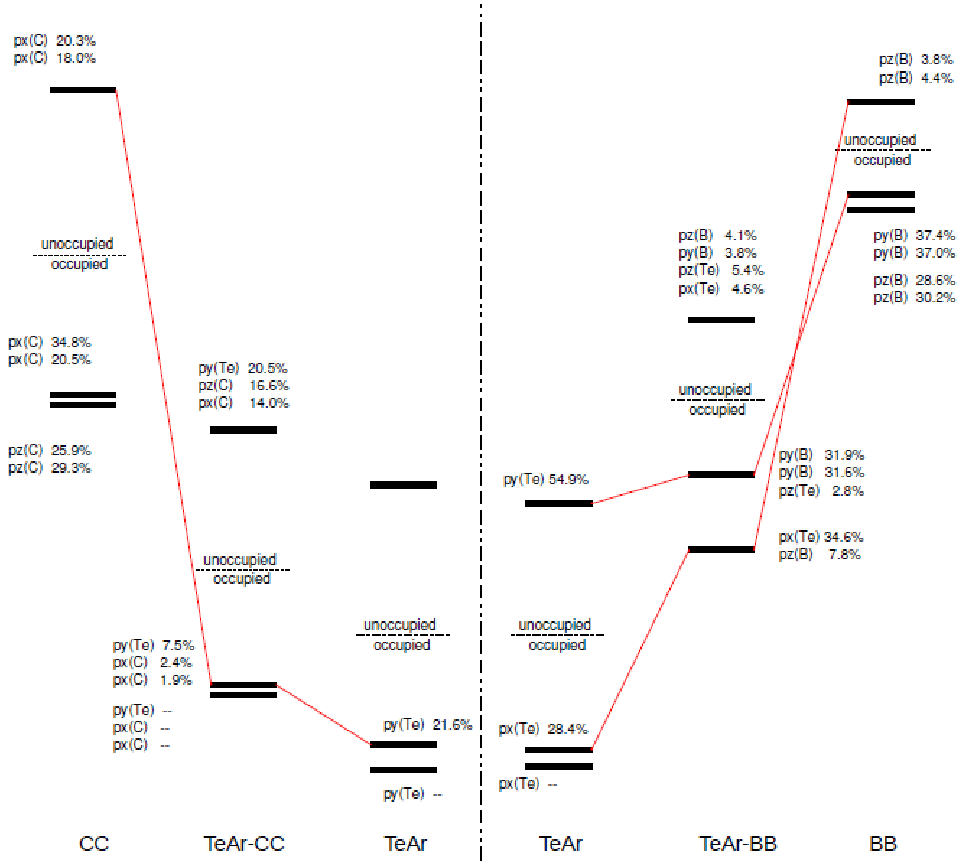

Figure S3. Energy level diagrams for the cationic portion of **1** and (tBu$_2$C$_2$)-TePh$^+$.

References

[1] Gaussian 09, Revision A.01, M. J. Frisch, G. W. Trucks, H. B. Schlegel, G. E. Scuseria, M. A. Robb, J. R. Cheeseman, G. Scalmani, V. Barone, G. A. Petersson, H. Nakatsuji, X. Li, M. Caricato, A. Marenich, J. Bloino, B. G. Janesko, R. Gomperts, B. Mennucci, H. P. Hratchian, J. V. Ortiz, A. F. Izmaylov, J. L. Sonnenberg, D. Williams-Young, F. Ding, F. Lipparini, F. Egidi, J. Goings, B. Peng, A. Petrone, T. Henderson, D. Ranasinghe, V. G. Zakrzewski, J. Gao, N. Rega, G. Zheng, W. Liang, M. Hada, M. Ehara, K. Toyota, R. Fukuda, J. Hasegawa, M. Ishida, T. Nakajima, Y. Honda, O. Kitao, H. Nakai, T. Vreven, K. Throssell, J. A. Montgomery, Jr., J. E. Peralta, F. Ogliaro, M. Bearpark, J. J. Heyd, E. Brothers, K. N. Kudin, V. N. Staroverov, T. Keith, R. Kobayashi, J. Normand, K. Raghavachari, A. Rendell, J. C. Burant, S. S. Iyengar, J. Tomasi, M. Cossi, J. M. Millam, M. Klene, C. Adamo, R. Cammi, J. W. Ochterski, R. L. Martin, K. Morokuma, O. Farkas, J. B. Foresman, and D. J. Fox, Gaussian, Inc., Wallingford CT, 2016.

[2] a) G. te Velde, F. M. Bickelhaupt, E. J. Baerends, C. Fonseca Guerra, S. J. A, van Gisbergen, J. G. Snijders, T. Ziegler, *J. Comput. Chem.* **2001**, *22*, 931−967; b) Amsterdam Density Functional, Theoretical Chemistry, Vrije Universiteit, Amsterdam, The Netherlands, http://www.scm.com.

[3] E. D. Glendening, J. K. Badenhoop, A. E. Reed, J. E. Carpenter, J. A. Bohmann, C. M. Morales, F. Weinhold, *NBO 5.0*; Theoretical Chemistry Institute, University of Wisconsin, Madison, WI, 2001.

[4] E. D. Glendening, J. K. Badenhoop, A. E. Reed, J. E. Carpenter, J. A. Bohmann, C. M. Morales, C. R. Landis, F. Weinhold *NBO 6.0*; Theoretical Chemistry Institute, University of Wisconsin, Madison, WI, 2013.

[5] F. A. Perras, W. C. Ewing, T. Dellermann, J. Böhnke, S. Ullrich, T. Schäfer, H. Braunschweig, D. L. Bryce, *Chem. Sci*. **2015**, *6*, 3378–3382.

[6] N. C. Handy, A. J. Cohen, *Mol. Phys.* **2001**, *99*, 403–412.

[7] D. P. Chong, *Mol. Phys.* **2005**, *103*, 749–761.

[8] D. P. Chong, E. van Lenthe, S. J. A. van Gisbergen, E. J. Baerends, *J. Comp. Chem.* **2004**, *25*, 1030–1036.

[9] E. van Lenthe, E. J. Baerends, *J. Comp. Chem.* **2003**, *24*, 1142–1156.

[10] R. C. Raffenetti, *J. Chem. Phys.* **1973**, *59*, 5936–5949.

[11] A. Berces, R. M. Dickson, L. Fan, H. Jacobsen, D. Swerhone, T. Ziegler. *Comp. Phys. Comm.* **1997**, *100*, 247–262.

[12] H. Jacobsen, A. Berces, D. Swerhone, T. Ziegler. *Comp. Phys. Comm.* **1997**, *100*, 263–276.

[13] S. K. Wolff. *Int. J. Quantum Chem.* **2005**, *104*, 645–659.

[14] I. Mayer. *Chem. Phys. Letts.* **1983**, *97*, 270–274.

[15] E. P. Fowe, B. Therrien, G. Süss-Fink, C. Daul, *Inorg. Chem.* **2008**, *47*, 42–48.

[16] F. L. Hirshfeld, *Theor. Chim. Acta*, **1993**, *44*, 129.

[17] K. B. Wiberg, P. R. Rablen, *J. Comp. Chem.* **1993**, *14*, 1504–1518.

[18] F. M. Bickelhaupt, E. J. Baerends. Kohn-Sham Density Functional Theory: Predicting and Understanding Chemistry. In K. B. Lipkowitz and D. B. Boyd, editors, *Reviews in Computational Chemistry*. Wiley-VCH, New York, 2000.

[19] K. Morokuma, *Acc. Chem. Res.* **1977**, *10*, 294–300.

[20] T. Ziegler, A. Rauk, E. J. Baerends, *Theo. Chim. Acta*, **1977**, *43*, 261–271.

[21] M. Mitoraj, A. Michalak, T. Ziegler, *J. Chem. Theo. Comp.* **2009**, *5*, 962–975.

[22] M. Mitoraj, A. Michalak, T. Ziegler, *Organometallics*, **2009**, *28*, 3727–3733.
